# Selenium status in term neonates, according to birth weight and gestational age, in relation to maternal hypertensive pathology

**DOI:** 10.3389/fped.2023.1157689

**Published:** 2023-03-30

**Authors:** Teofana Otilia Bizerea-Moga, Laura Pitulice, Otilia Bizerea-Spiridon, Claudiu Angelescu, Otilia Mărginean, Tudor Voicu Moga

**Affiliations:** ^1^ Department XI of Pediatrics-1st Pediatric Discipline, Center for Research on Growth and Developmental Disorders in Children, “Victor Babeș” University of Medicine and Pharmacy Timișoara, Timișoara, Romania; ^2^ 1st Pediatric Clinic, “Louis Țurcanu” Children’s Clinical and Emergency Hospital, Timișoara, Romania; ^3^ Department of Biology-Chemistry, West University of Timişoara, Timişoara, Romania; ^4^ Laboratory of Advanced Researches in Environmental Protection, Timişoara, Romania; ^5^ Clinic of Obstetrics, Gynecology and Neonatology, “Pius Brînzeu” County Emergency Clinical Hospital, Timișoara, Romania; ^6^ Department VII of Internal Medicine-Gastroenterology Discipline, Advanced Regional Research Center in Gastroenterology and Hepatology, “Victor Babeș” University of Medicine and Pharmacy Timișoara, Timișoara, Romania; ^7^ Gastroenterology and Hepatology Clinic, “Pius Brînzeu” County Emergency Clinical Hospital, Timișoara, Romania

**Keywords:** pregnancy-induced hypertension, oxidative stress, selenium, neonate, gestational age, birthweight

## Abstract

**Background:**

Pregnancy represents a state of increased oxidative stress and antioxidants, in which selenium (Se) plays a pivotal role, contribute to maintain the oxidative balance. If antioxidant defenses are depleted, placental function is disrupted, resulting in pregnancy complications, including pregnancy-induced hypertension (PIH). Little is known about fetal selenium status in concomitant relation to maternal PIH, gestational age (GA) and birthweight (BW).

**Methods:**

We examined over a 3-year period the serum (SeS) and urine selenium (SeU) status in term neonates from normotensive (nonPIH) and hypertensive (PIH) mothers as clinical markers of oxidative stress. In this retrospective observational study, 72 neonates with maternal PIH were matched for GA and BW to 72 neonates of normotensive mothers. Four groups were obtained, based on maternal PIH and BW relative to GA (appropriate-for-gestational-age—AGA, small-for-gestational-age—SGA): nonPIH-AGA (control group), nonPIH-SGA, PIH-AGA, and PIH-SGA.

**Results:**

The results showed significant differences (*p* < 0.001) in selenium levels among the study groups: SeS - 44.85 ± 7.56 μg/L in nonPIH-AGA, 39.62 ± 11.42 μg/L in nonPIH-SGA, 40.01 ± 10.07 μg/L in PIH-AGA, and 25.39 ± 8.99 μg/L in PIH-SGA; SeU - 27.98 ± 7.99 μg/L in nonPIH-AGA, 22.85 ± 9.48 μg/L in nonPIH-SGA, 23.44 ± 6.73 μg/L in PIH-AGA, and 13.05 ± 5.86 μg/L in PIH-SGA. Selenium depletion was more common in neonates born from hypertensive mothers and those born small for gestational age. Though moderate in intensity, selenium levels were positively correlated with BW (0.319 for SeS, 0.397 for SeU) and negatively correlated with maternal systolic blood pressure (−0.313 for SeS, −0.324 for SeU). The main independent effects on SeS and SeU of each maternal blood pressure and birth weight turned out statistically significant. In interaction, a more pronounced effect was reached in PIH-SGA neonates.

**Conclusion:**

Selenium status seemed to reflect the negative impact that PIH exerts in neonates during intrauterine development. Clinical markers of selenium status could thus be of great value for tracking responses of individuals to selenium supplementation as part of health improvement and harm mitigation approaches.

## 1. Introduction

Fetal development and perinatal survival rely on the proper function of the maternal–placental–fetal unit. A critical component altering this equilibrium is oxidative stress (OS), consisting in the disturbance of the antioxidant defense system. Extensive studies in the literature have acknowledged that selenium plays a crucial role in the biological functions of the human organism. Antioxidant defense represents selenium's key function, which is closely related to human fertility and reproduction (1). Pregnancy itself is a special condition of OS due to the increased demand for oxygen both in mother and fetus, and due to the generation of reactive oxygen species (ROS) at the placental level (2–4).

Selenium (Se), through selenoproteins, is implicated in regulation of ROS levels and redox equilibrium in almost all body tissues. Selenoproteins are particularly well-suited for combating OS due to their unique redox structural motifs (5). In depth, combined analysis of protein conformational preferences and mobilities change (6–8) could explain the way the presence of selenium and variations of the confined environment can modulate the motifs' reactivity—rendering selenoproteins sensitive to low levels of oxidants and resilient to inactivation by the ROS (5). However, the antioxidant activity genetically programed during pregnancy may be insufficient to compensate for ROS excess. As such, a disrupted antioxidant system, mainly revealed as a selenium deficiency, is responsible for a variety of pregnancy complications, including pregnancy-induced hypertension (PIH) (9), preterm birth (10, 11), intrauterine growth restriction (IUGR) (12) and delivery of small-for-gestational-age (SGA) infants (13). Maternal hypertensive pathologies, associating a selenium-reduced status, represent, in turn, a significant factor affecting antioxidant system imbalance in fetuses which can have significant repercussions on fetal development. In fact, literature findings reveal a two-way association between PIH and OS: on the one hand, pregnancy generates OS that in turn is the main contributor to the etiology of hypertensive disorders of pregnancy, including PIH (14, 15); on the other hand, PIH is a state of extremely increased oxidative stress, put into evidence experimentally by different OS markers (14, 16, 17).

There is a strong body of evidence indicating a correlation between selenium status in mothers, birthweight and gestational age of neonates born from mothers with different pathologies, including PIH (9, 13, 18–26). It is also widely acknowledged that PIH influences fetal development. There are some studies evaluating selenium levels in neonates, and mostly in preterm, in close relation to maternal selenium levels as a basis for optimization of selenium supplementation (20, 27–30). However, little is known about selenium levels in term neonates in concomitant relation to maternal PIH, gestational age, and birthweight.

Neonatal Se levels are not routinely measured as standard of care (31). However, among biomarkers used to assess Se status, blood and urine Se easily translate into use in neonate clinical settings. Blood contains almost half of the body's selenium, and blood Se concentration properly reflects Se intake or exposure; whole blood and erythrocyte Se reflect long-term status, while the serum/plasma Se (SeS) indicates short-term status (32, 33). Urine, on the other hand, is the main route of Se excretion (34, 35) with a selenosugar (1*β*-methylseleno-N-acetyl-D-galactosamine) being the predominant speciation (34). Thus, urinary Se (SeU) concentration can also be used to assess Se status (36) and 24-h urine collection seems to be the most appropriate method for measurement (32).

The present study was aimed at:

 - assessing the variations of serum and urinary Se levels among term newborns from normo- and hypertensive mothers;
 - revealing if altered Se levels in term neonates affected by the oxidative challenges manifested in maternal PIH state correlate with maternal hypertension, BW and GA, making thus Se level a suitable clinical marker;
 - identifying the extent to which neonates might be affected by PIH, i.e., how pronounced their Se deficiency is compared to healthy term neonates born from normotensive mothers.

The correlation of Se levels in response to maternal PIH could provide insights into the mechanism by which PIH determines worse outcomes in neonatal development.

## 2. Materials and methods

### 2.1. Operational definitions

Case-mix records were used to identify the cases of maternal pregnancy-induced hypertension, using the Romanian Diagnosis-Related Group classification system version 1 (RO DRG v1): code O13.0 (gestational hypertension).

Newly born babies whose birth weight (BW) was less than two standard deviations (<-2SD) or the 10th percentile (< Perc.10%) below the mean for gestational age (GA) were included as SGA (37).

### 2.2. Study design

This retrospective observational study included neonates born from normotensive and hypertensive mothers over a 3-year period, from the 1st of January 2017 until the 31st of December 2019, at the Obstetrics, Gynecology and Neonatology Clinic of the Emergency County University Hospital “Pius Brinzeu” in Timişoara, Romania. The study followed the guidelines of the Helsinki Declaration regarding the use of human subjects. The Ethic Committee for Research of the “Victor Babeș” University of Medicine and Pharmacy from Timişoara approved sharing of anonymized patient data. Individual consent to collect blood and urine samples from the newborns for Se analysis, was obtained from caregivers at the time of hospital admission. The data were collected retrospectively from archived records of in-hospital born patients in the Department of Neonatology.

During the study period, 84 neonates born from mothers with PIH were identified in the archive files of the Neonatology Department. Neonates' selection was conducted according to the following exclusion criteria: maternal sepsis, maternal causes for placental insufficiency other than PIH, perinatal asphyxia and neonatal sepsis, and genetic disorders. Accordingly, 12 neonates were excluded from the study.

**Figure 1:**
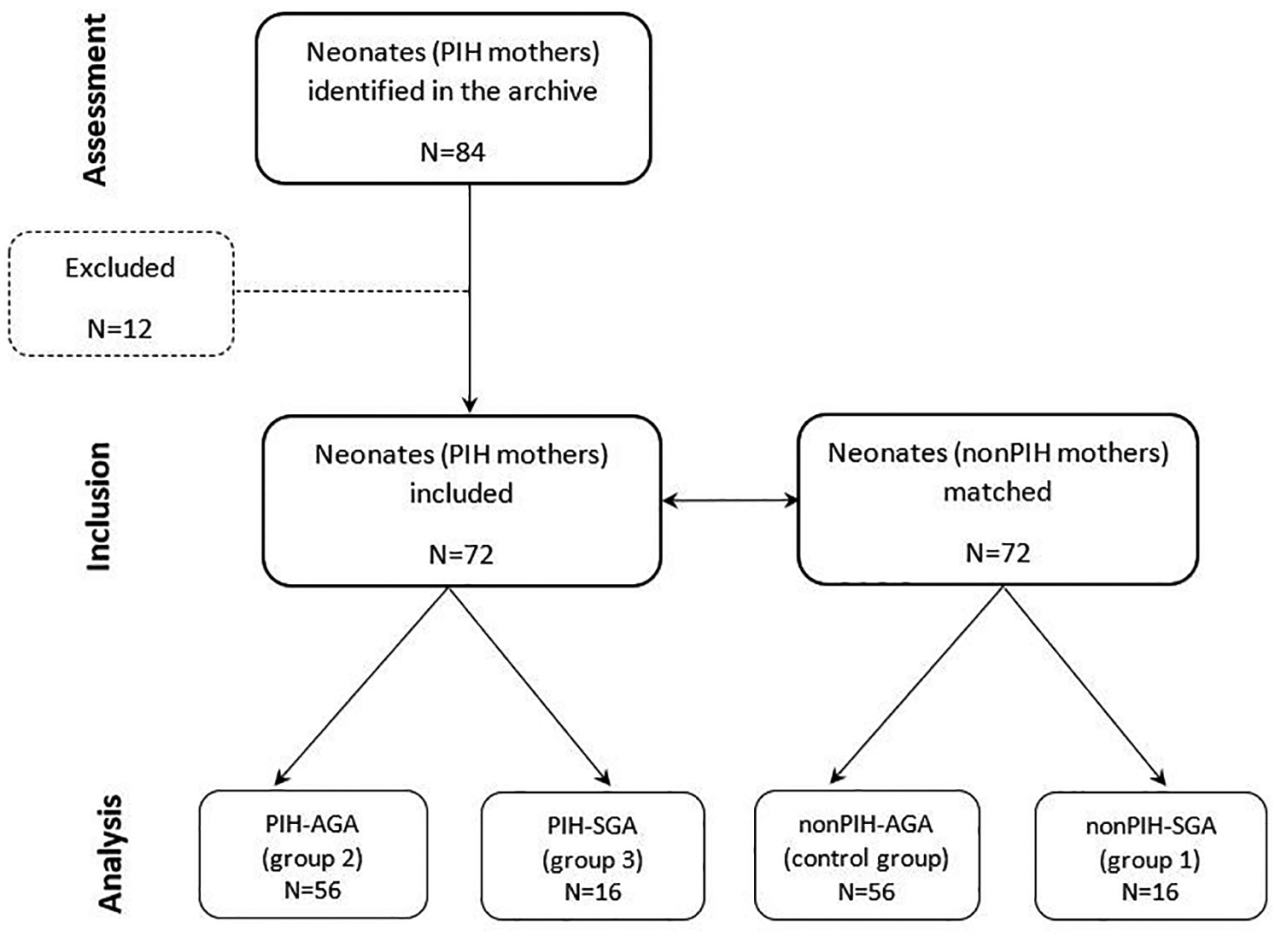
CONSORT diagram of study design.

The 72 neonates with maternal PIH included in the study were matched for gestational age and birth weight to 72 neonates of normotensive mothers (nonPIH). As such, for each appropriate-for-gestational-age (AGA) neonate with maternal PIH, a corresponding AGA neonate born to a nonPIH mother was chosen for the study. This pairing procedure was also used for SGA neonates of mothers with and without PIH, respectively. Three study groups were formed according to birth weight for gestational age and presence or absence of maternal PIH. The first study group (nonPIH-SGA) included 16 SGA neonates from nonPIH mothers. The second study group (PIH-AGA) included 56 AGA neonates with maternal PIH. The third study group (PIH-SGA) included 16 SGA neonates with maternal PIH. Fifty-six AGA neonates from nonPIH mothers were included in the control group (nonPIH-AGA). The study design is included in Figure 1.

### 2.3. Selenium analysis

The investigation of Se status in newly born babies was part of an EU-funded project (Strategic partnership for the enhancement of scientific research of medical universities by awarding doctoral and postdoctoral scholarships-DocMed.Net_2.0, POSDRU/159/1.5/S/136893) that was implemented by the “Victor Babeș” University of Medicine and Pharmacy from Timișoara during the 3-year study period. Se status was evaluated based on its concentrations in serum and urine. Venous blood samples and urine samples obtained in the first 24 h of life of the neonates were collected at the Obstetrics, Gynecology and Neonatology Clinic of the Emergency County University Hospital “Pius Brînzeu” and sent to an independent, offsite laboratory in Timișoara for analysis. The Bioclinica Medical Analysis Centre (referred to as Bioclinica) is one of the top-rated private medical laboratories in Romania and is headquartered in Timișoara. Pursuant to a collaboration agreement with “Victor Babeș” University of Medicine and Pharmacy from Timișoara, Bioclinica provided services for the analysis of microelements from human fluids. Se analyses were performed using an inductively coupled plasma mass spectrometry (ICP-MS) method, as described elsewhere (33).

### 2.4. Statistical approach

Statistical processing of data was performed using SPSS version 26.0 (IBM, Armonk, NY). The data collected from the study subjects were verified and doubly entered into a data management system. Normally distributed data were presented as means and standard deviation (SD) and compared by Student's t-test and one way ANOVA, whereas non-normally distributed data were presented as medians and interquartile ranges (IQR), defined by 25 and 75 percentiles, and compared by Mann–Whitney U test or Kruskal–Wallis test. Categorical variables were presented by absolute frequency and percentage of the sample and compared by chi-squared test. Factorial ANOVA was used to investigate the main effects and interactions of independent variables and post-hoc Bonferroni test was employed for manifested differences between each two groups. Spearman correlations were used to explore relationships between data. Two-sided *p*-values < 0.05 were considered statistically significant.

## 3. Results

In this study, Se status was assessed by measuring its concentration in serum and urine samples of neonates. The correlation between Se status, BW, and maternal hypertensive pathology was studied, on one hand, through variations of Se concentration with BW in neonates from normotensive and hypertensive (nonPIH and PIH) mothers. On the other hand, the correlation was studied through variations of Se concentration with maternal blood pressure (BPmat) in normal and small-for-gestational-age (AGA and SGA) neonates.

A total of 144 neonates were included in the study, comprising 72 neonates of hypertensive mothers paired with 72 neonates of normotensive mothers. The demographic characteristics and clinical data of the neonates of each study group along with the significance threshold value (*p*) of differences between all groups are presented in Table 1.

**Table 1:** The clinical and biological characteristics of each study group.

Parameters	Control Group nonPIH-AGA (*N* = 56)	Study Group 1 nonPIH-SGA (*N* = 16)	Study Group 2 PIH-AGA (*N* = 56)	Study Group 3 PIH-SGA (*N* = 16)	*N* = 144	*p*
Gender						0.485*
Male	24 (42.9%)	6 (37.5%)	26 (46.4%)	10 (62.5%)	66 (45.8%)	
Female	32 (57.1%)	10 (62.5%)	30 (53.6%)	6 (37.5%)	78 (54.2%)	
Birth						0.223*
Natural	20 (35.7%)	5 (31.3%)	11 (19.6%)	3 (18.8%)	39 (27.1%)	
Cesarean	36 (64.3%)	11 (68.7%)	45 (80.4%)	13 (81.2%)	105 (72.9%)	
Maternal Systolic BP	110.00 (105.00–115.00)	109.00 (102.25–114.75)	146.00 (142.00–153.75)	160.00 (155.00–173.75)	127.50 (110.00–150.00)	<0.001**
BW (g)	3382.50 (3057.50–3626.25)	2325.00 (1947.50–2465.00)	3340.00 (3105.00–3750.00)	2444.00 (2090.00–2677.50)	3212.50 (2676.25–3608.75)	<0.001**
GA (weeks)	39.00 (38.00–40.00)	38.00 (37.00–39.00)	38.00 (38.00–39.00)	38.00 (37.00–39.00)	39.00 (38.00–39.00)	0.006**
Birth Length (cm)	50.00 (49.00–52.00)	47.00 (46.00–48.00)	52.00 (50.00–53.00)	49.00 (46.25–49.75)	50.00 (48.00–52.00)	<0.001**
Head Circumference (cm)	34.00 (33.00–35.00)	32.00 (31.25–33.00)	34.00 (33.00–35.75)	33.00 (31.25–33.00)	34.00 (33.00–35.00)	<0.001**
APGAR Score	9.00 (8.00–9.75)	8.00 (7.00–9.00)	9.00 (8.00–9.00)	8.50 (8.00–9.00)	9.00 (8.00–9.00)	0.005**
SeS (μg/L)	44.85 ± 7.56	39.62 ± 11.42	40.01 ± 10.07	25.39 ± 8.99	40.22 ± 10.78	<0.001***
SeU (μg/L)	27.98 ± 7.99	22.85 ± 9.48	23.44 ± 6.73	13.05 ± 5.86	23.98 ± 8.65	<0.001***
% SeU / SeS	61.62 ± 11.20	59.79 ± 21.26	59.18 ± 12.10	52.83 ± 15.91	59.49 ± 13.62	0.155***

Categorical variables are presented by absolute frequency and percentage of the sample (*chi-squared test); continuous variables with non-Gaussian distribution are indicated by their median and 1st and 3rd quartile values (**Kruskal Wallis test); Gaussian-distributed continuous data are depicted as mean ± standard deviation (***one way ANOVA test).

Regarding the demographic data, there were no significant differences between neonates from mothers with PIH and those from healthy mothers regarding gender and, most surprisingly, APGAR scores at birth. Overall, up to three-quarters of all births were by cesarean—a procedure that was more likely performed in hypertensive mothers and in those with SGA offspring. One of the main reasons for the high rate of cesarean sections (C-sections) in women with PIH is the increased risk of cardiovascular complications after delivery (e.g., postpartum stroke), chronic hypertension and mood disorders (e.g., depression anxiety) (38). Another reason is the need to control blood pressure during delivery. In some cases, medications may be used to lower blood pressure, but these can have side effects on the mother and the baby. Therefore, C-sections may be preferred to avoid the stress of labor, which can further increase blood pressure. Noteworthy, C-section rate in Romania (44.1% in 2017) was estimated by the Eurostat as the second highest in the European Union (39), reaching as high as 80% in private clinics (40). C-section rates of 60.7% (41) and 64% (42) were found in two studies performed in two large maternity hospitals in Romania. It is also acknowledged that nowadays in Romania more women elect C-section delivery regardless of a medical recommendation based on prejudgments, fear, or misbeliefs amplified by low level of medical understanding (40–42).

The anthropometric measurements of birth length and head circumference were similar among the groups. Additionally, there was no indication of significant influence of PIH on neonates’ birth lenght and GA. However, PIH influence on SeS and SeU concentrations was clearly noted, mostly for SGA neonates, illustrating the enhanced oxidative stress present in PIH state.

### 3.1. Selenium status in relation to oxidative stress

The first part of the present study focused on comparing the neonates' Se status as an expression of the state of their defense system against the intrauterine OS. Therefore, SeS and SeU variations were analyzed between the studied groups and compared to the control group. Levene's test was used to assess the homogeneity of variance, and t-test for independent samples was utilized to compare the mean values of serum and urine selenium between the studied groups. Moreover, to explore the difference between each two groups the post-hoc Bonferroni test was employed.

The mean SeS level in the control group was 44.85 ± 7.56 μg/L, while in the study groups, it was 39.62 ± 11.42 μg/L in nonPIH-SGA, 40.01 ± 10.07 μg/L in PIH-AGA, and 25.39 ± 8.99 μg/L in PIH-SGA, respectively. Thus, significantly (*p* < 0.001, Table 1) higher values of Se were found in neonates from the control group than in those from the study groups.

The highest levels of SeU were found in nonPIH-AGA neonates of the control group, with a mean value of 27.98 ± 7.99 μg/L, compared to 22.85 ± 9.48 μg/L in the nonPIH-SGA group, 23.44 ± 6.73 μg/L in the PIH-AGA group, and 13.05 ± 5.86 μg/L in the PIH-SGA group (*p* < 0.001, Table 1).

In PIH-SGA neonates, the lowest average levels of Se were observed, significantly (*p* < 0.001) lower than in nonPIH-SGA neonates and in PIH-AGA neonates, respectively, regarding both SeS (25.39 ± 8.99 μg/L compared to 39.62 ± 11.42 μg/L and 40.01 ± 10.07 μg/L), and SeU (13.05 ± 5.86 μg/L, 22.85 ± 9.48 μg/L and 23.44 ± 6.73 μg/L, respectively).

Statistically significant differences, demonstrated by values of *p* < 0.05 regarding SeS and SeU levels in neonates from the studied groups, are shown in Table 2.

**Table 2:** Comparison of SeS and SeU values between the study groups and between each study group and the control group (nonPIH-AGA).

Group Statistics*				
Selenium parameter		*nonPIH-AGA*	*nonPIH-SGA*	*PIH-AGA*
	Study Groups		*p*	
SeS (μg/L)	*nonPIH-SGA*	0.034		
	*PIH-AGA*	0.005	0.893	
	*PIH-SGA*	<0.001	<0.001	<0.001
SeU (μg/L)	*nonPIH-SGA*	0.033		
	*PIH-AGA*	0.002	0.779	
	*PIH-SGA*	<0.001	0.001	<0.001

* Correlation is significant at the 0.05 level (2-sided).

Similar mean Se values were found in the nonPIH-SGA and the PIH-AGA study groups, both in terms of SeS and SeU, with no statistically significant differences (39.62 ± 11.42 μg/L compared to 40.01 ± 10.07 μg/L and 22.85 ± 9.48 μg/L, respectively, 23.44 ± 6.73 μg/L).

To further deepen the analysis and explore the main independent effects of blood pressure and birth weight and their pre-specified interaction on Se levels, the factorial ANOVA was performed.

The results indicated that both BPmap and BW have a statistically significant effect on SeS [F(1,143) = 26.62, F(1, 143) = 28.88, *p* < 0.05], as well as their combined interaction [F(1, 143) = 6.45; *p* = 0.012]. Particularly, an increased level of blood pressure leads to a decrease of SeS level. Also, a low birth weight results in lower SeS levels. In interaction, the BPmat effect on SeS depends on the neonate's BW, with a more pronounced effect reached in PIH-SGA neonates.

The main effects on SeU of each BPmat and BW also turned out statistically significant [F(1,143) = 22.76, F(1, 143) = 26.67, *p* < 0.05] but not their synergistic interaction [F(1, 143) = 3.06; *p* = 0.082].

### 3.2. Selenium status and birth weight

**Figure 2:**
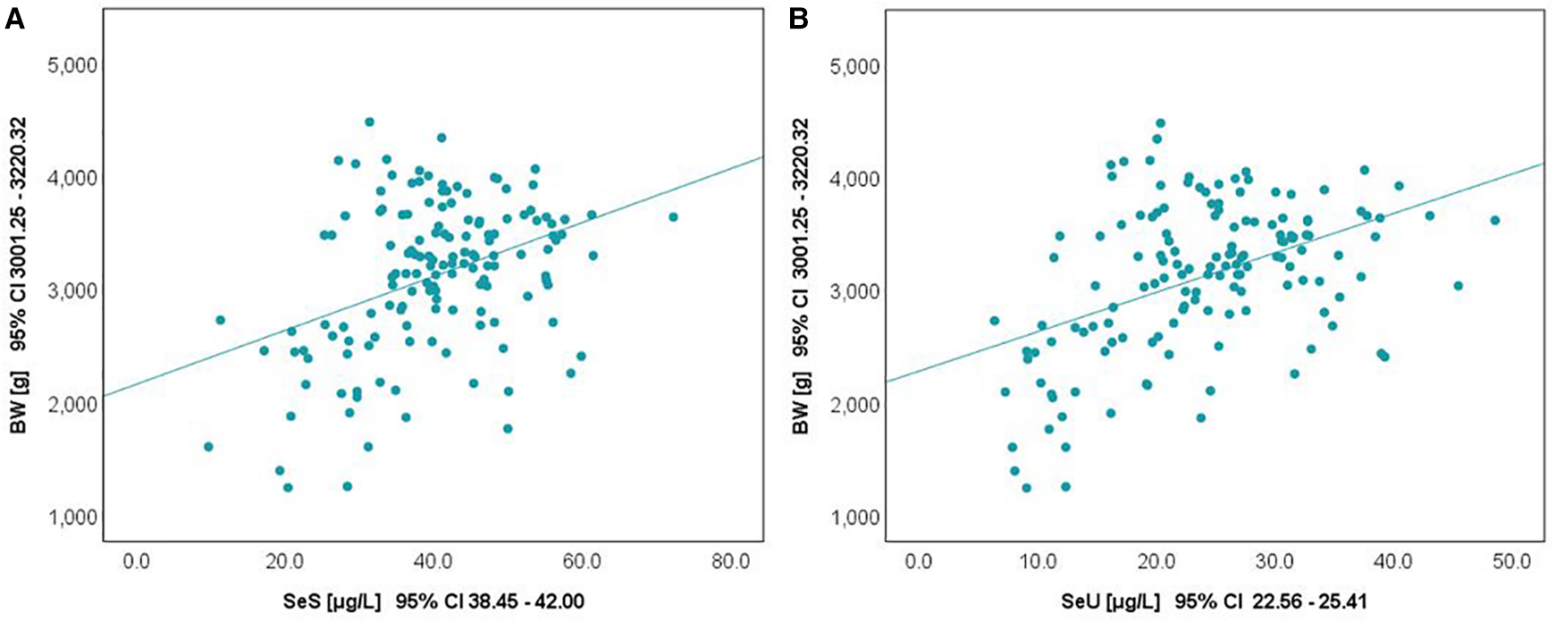
Variation of SeS (**A**) and SeU (**B**) with BW in neonates from all study groups.

The relationship between selenium levels and birth weight was also studied. Spearman correlation (significant at the 0.01 level, 2-tailed) was used to examine the relationship between Se levels and BW values. The results are depicted in Figure 2. Positive correlations between the analyzed parameters were observed in both cases. The value of the correlation coefficient, Spearman's rho (rS), expressed the intensity of the relationship between variables. In this case, an rS value between 0.3 and 0.5 showed a moderate correlation.

**Figure 3:**
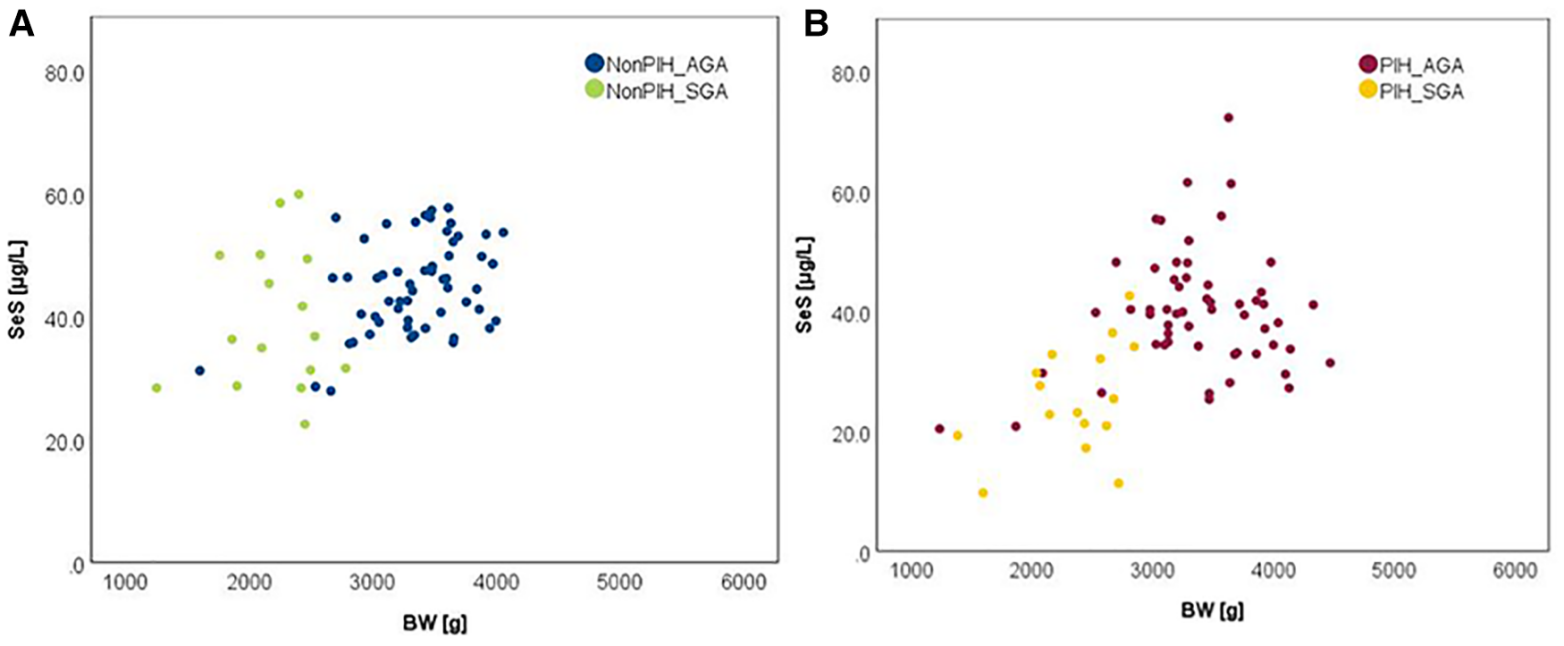
Variation of SeS with BW in neonates from normotensive mothers (**A**) and hypertensive mothers (**B**).

**Figure 4:**
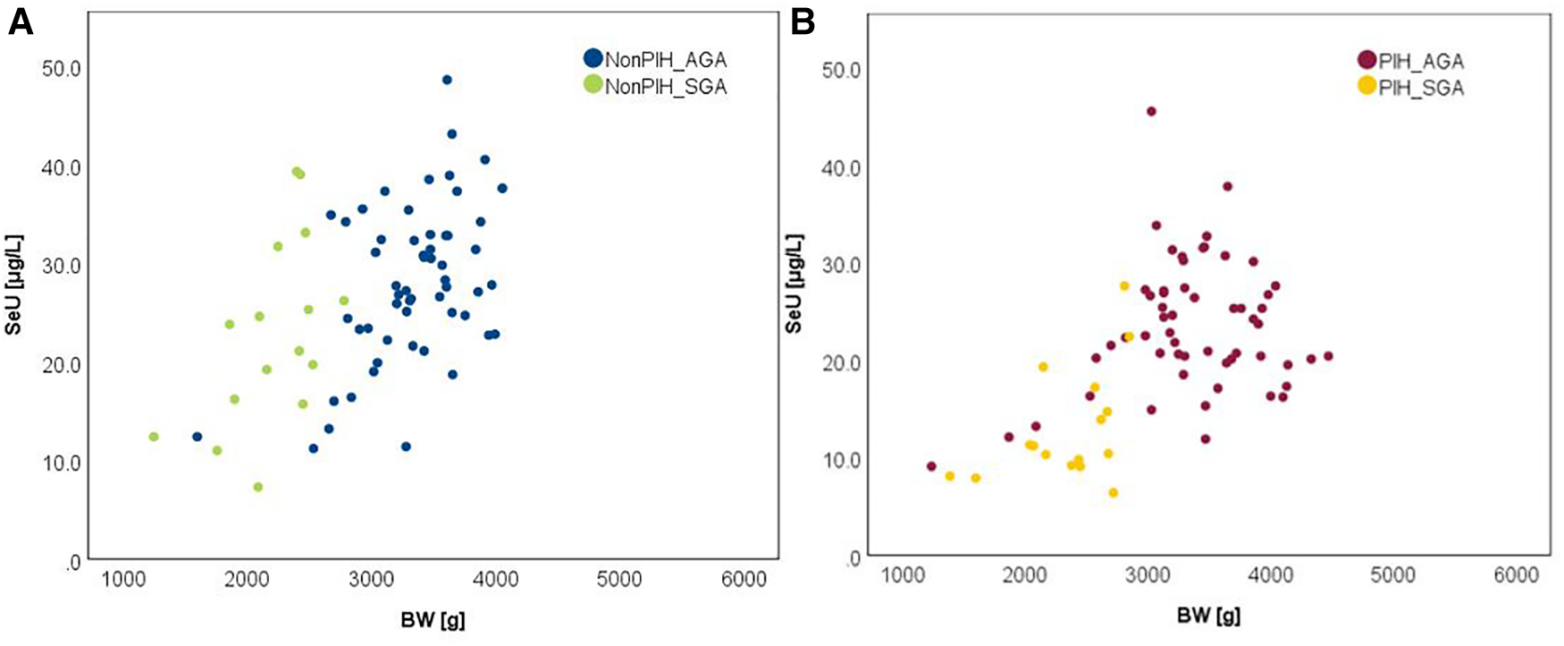
Variation of SeU with BW in neonates from normotensive mothers (**A**) and hypertensive mothers (**B**).

The influence of the augmented oxidative stress that is present in maternal PIH on fetal development and birthweight is shown in Figures 3, 4, using Se status as a clinical marker. A negative influence of maternal hypertensive pathology on neonatal birthweight was observed. Clearly, SGA neonates were the most affected by PIH, illustrated by lower Se levels. However, in the nonPIH-SGA group, the Se values were quite dispersed around the central tendency (SeS: 39.62 ± 11.42 μg/L and SeU: 25.39 ± 8.99 μg/L), and in the PIH-SGA group Se values tended to concentrate into a narrow, though lower, interval (SeS: 22.85 ± 9.48 and SeU: 13.05 ± 5.86).

### 3.3. Selenium status and pregnancy induced hypertension

**Figure 5:**
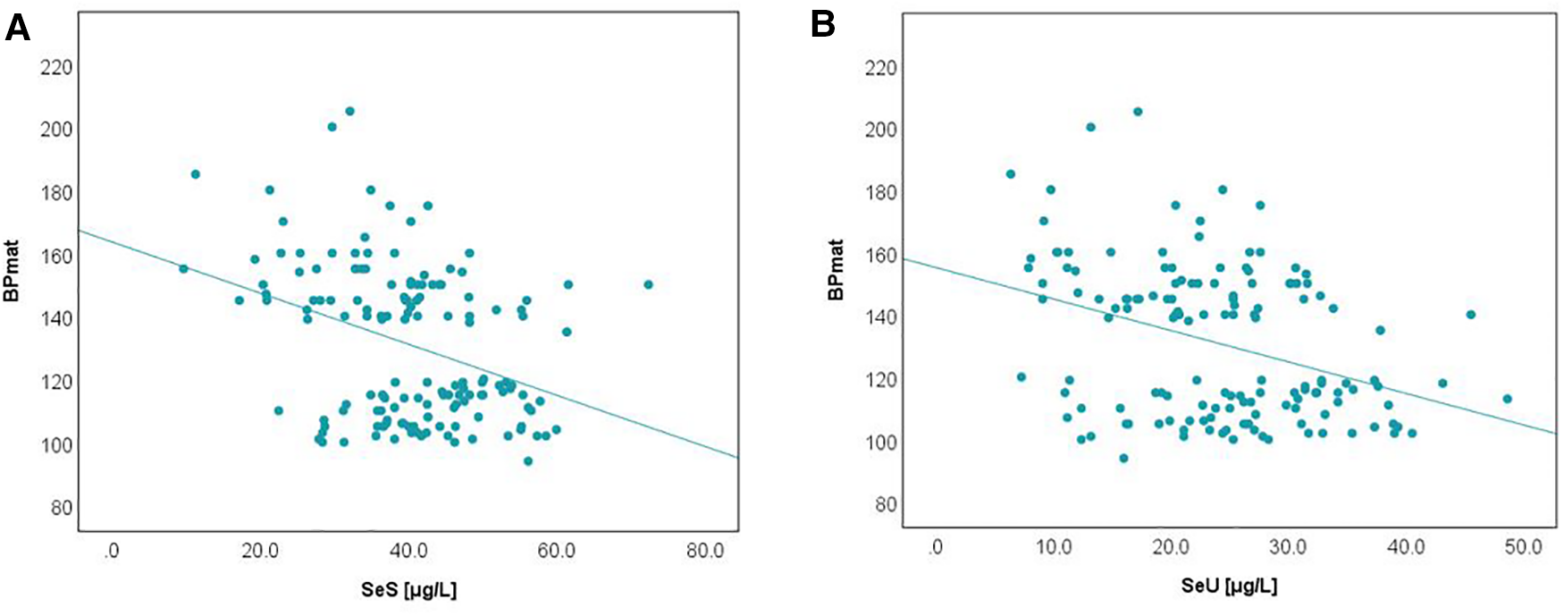
Variation of SeS (**A**) and SeU (**B**) with BPmat in neonates from all study groups.

It has been recognized that maternal PIH represents a state of an extremely increased OS, but its relationship with term neonates' selenium status calls for further investigation. The bivariate correlation analysis, based on Spearman's rho, reflected a negative, moderate intensity correlation between both SeS and SeU and BPmat, i.e., low SeS and SeU values were associated with increased systolic BPmat values. These results are depicted in Figure 5.

**Figure 6:**
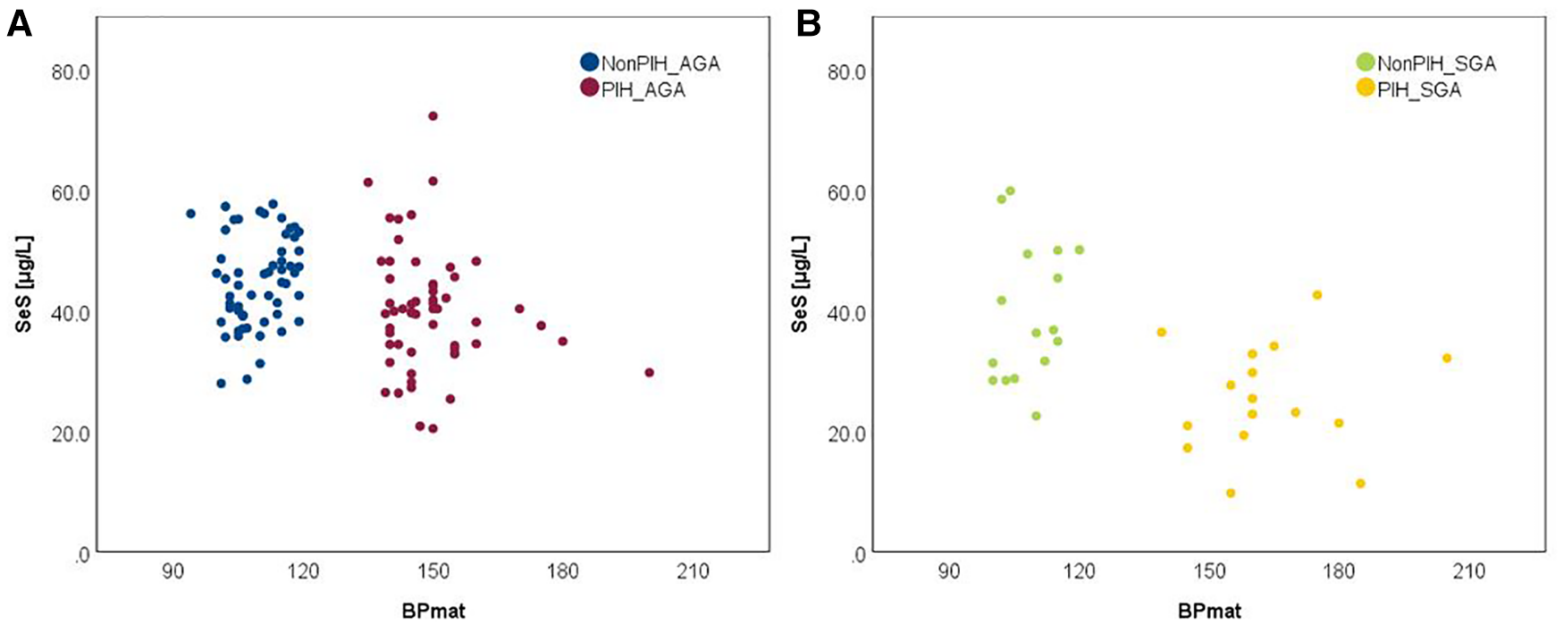
Variation of SeS with BPmat in AGA neonates (**A**) and SGA neonates (**B**).

**Figure 7:**
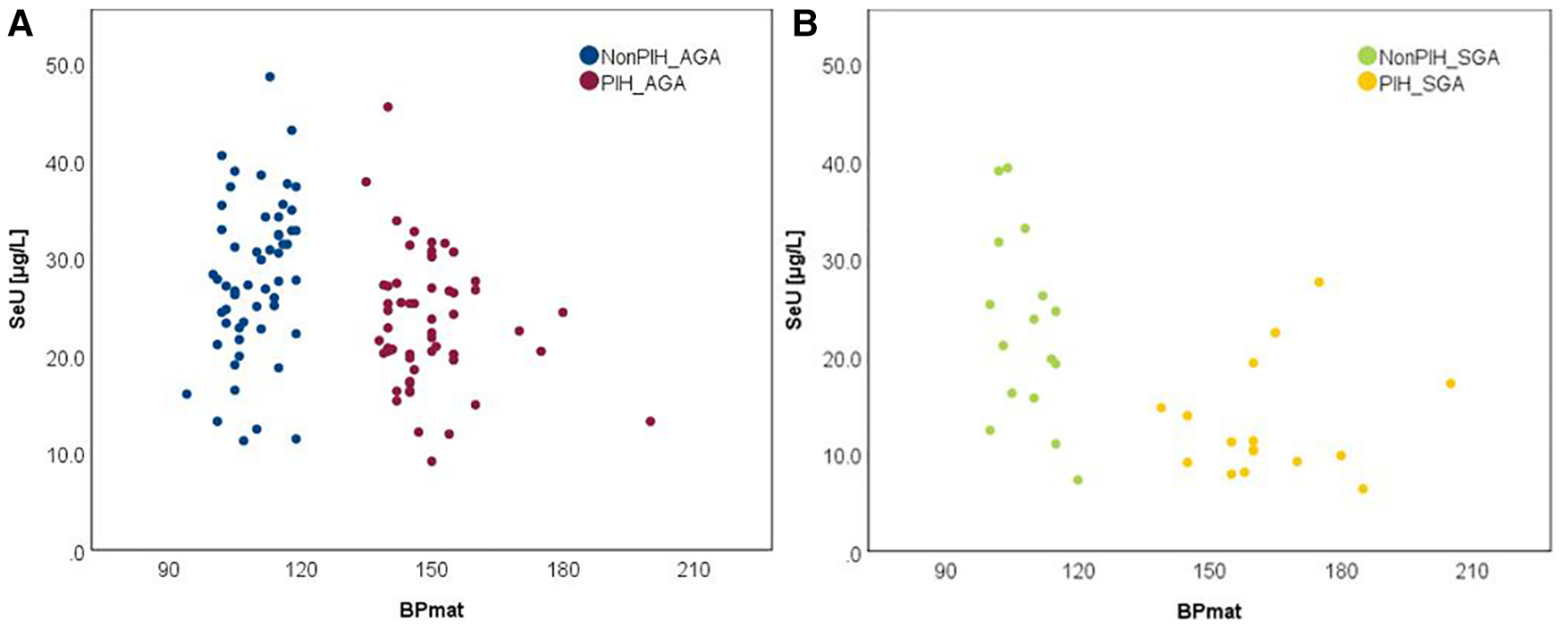
Variation of SeU with BPmat in AGA neonates (**A**) and SGA neonates (**B**).

Comparison of Se levels between the AGA and SGA groups better illustrated the impact of PIH, i.e., the PIH influence was more pronounced on SGA neonates (Figures 6, 7).

In each figure, there are two groups of neonates in terms of BPmat values that fall either into healthy (from nonPIH mothers) or pathological category (from PIH mothers). PIH is defined as systolic blood pressure (SBP) > 140 mmHg and diastolic blood pressure (DBP) > 90 mmHg (43). Thus, children whose mothers had borderline systolic blood pressure (between 121 and 139 mmHg) were not included in the study. The oxidative stress associated with a mild increase of blood pressure is most likely counterbalanced by compensatory antioxidative mechanisms, and observations regarding the link to Se-levels might be biased. Within the SPB buffer range of 121–139 mmHg it is possible that the antioxidant mechanisms can cope with the excess of free radicals and OS, and thus Se levels can be maintained at the optimal level. Consequently, an augmented OS might not be perceived through Se levels.

In order to detect the extent to which term neonates were affected by the intrauterine oxidative stress, we looked at differences in both serum and urinary Se levels of study groups against the control group, representing the reference values. We observed a similar decrease of the mean SeS values in nonPIH-SGA and PIH-AGA (11.66% and 10.79%) compared to nonPIH-AGA. However, in the PIH-SGA group the mean SeS values decreased by almost half (47.85%). While a similar tendency was seen for the mean SeU values, their decreases were even more pronounced (18.33%, 16.23% and 53.36%). Overall, PIH pathology is clearly associated with Se deficiency in term neonates.

## 4. Discussion

Our results showed that there were statistically significant differences in Se status at birth between AGA and SGA babies from healthy normotensive mothers, with both serum and urinary Se concentrations being lower in the latter. These observations agreed with those of other studies that evaluated the relationship between BW and Se status (20, 29, 44–51). While Strambi et al. noted no such differences at birth, it was later observed that serum Se levels began to decline in SGA when compared to AGA infants (19). Other research showed no correlation between SeS levels and gestational age and/or weight (52, 53). This situation could reflect the importance of Se in the adaptative response to OS during fetal intrauterine growth. Increased Se levels could play an important role in reducing the risk for SGA neonates and favoring an adequate intrauterine development.

Between the 20th and 40th weeks of gestation, Se is stored in the fetal liver. Because of intrauterine growth restriction, SGA babies are born with decreased hepatic Se stores. This, in turn, could further predispose them to Se deficiency in the neonatal period in which the rapid growth rate can lead to an increase in Se requirements (21, 24, 52, 54).

During the intrauterine growth period, selenium is indispensable for the developing fetus. The transport of selenium across the placenta occurs by passive diffusion according to a concentration gradient, which appears to work in both directions (24, 55–61). Therefore, the placenta acts as a regulator of Se transfer to the fetus (62). The concentration of Se in the umbilical cord blood of newly born babies represents 60%–70% of the maternal SeS levels (3, 9, 20, 24, 25, 63, 64). Two other mechanisms have been identified to contribute to maternal-fetal Se transfer involving the extracellular selenoproteins SELENOP (Sepp1) and plasma glutathione peroxidase (GPx3). On the one hand, the apolipoprotein E receptor-2 (ApoER2) expressed by the placenta, binds Sepp1 and mediates its endocytosis from the systemic circulation. On the other hand, bulk pinocytosis of Sepp1 and GPx3 supplies fetus with selenium *via* the uterine fluid (65).

SGA infants of healthy mothers, as well as AGA infants of mothers with PIH, were both exposed to OS during the intrauterine development period (15, 66, 67). Selenium is known to play an important antioxidant role; therefore suboptimal Se levels can lead to a weakened defense system against OS (68). In the studied patients, both SeS and SeU levels were lower than in the control group, but did not differ, in terms of statistical significance, between the two groups. This finding could be explained by the fact that neonates from both study groups were exposed to a certain degree of intrauterine OS that expressed differently. In SGA neonates from normotensive mothers, the impact of OS on intrauterine growth was quite intuitive. In case mothers were Se-deficient, this could have been transferred to their newborns (30). However, in the PIH-AGA study group, OS resulted from the imbalance induced by maternal hypertension.

Scarce data could be found in the literature on Se status of neonates with various disorders due to the increased OS of the maternal PIH. In our study, neonates with maternal PIH had significant lower SeS and SeU levels than neonates from nonPIH mothers. These findings were supported by other research. Mistry et al. found lower Se levels in umbilical venous samples in neonates from preeclamptic women, in comparison with samples from babies of normotensive mothers (*p* < 0.0001) (9). The lowest levels of selenium were found in SGA neonates with maternal PIH (*p* ≤ 0.001). PIH and SGA, because of intrauterine growth restriction, share similar pathophysiological features involving the placenta (69). PIH occurs because of shallow trophoblast invasion accompanied by inadequate remodeling of the spiral arteries, leading to placental insufficiency. Decreased placental perfusion generates a significant increase in ROS at this level. ROS, in turn, induce premature aging of the placenta and maintain placental insufficiency. This vicious cycle prevents fetal needs from being met by the placenta, resulting in intrauterine growth restriction (70, 71). Abnormal placental architecture and function, leading to placental insufficiency, might limit transplacental transfer of Se to the fetus with intrauterine growth restriction. This occurs due to immature chorionic villi that act in the transport of this element (9, 46). This observation reinforces the idea that Se is a reliable predictor of the impact of the augmented OS in maternal PIH on neonates and that the requirement for Se can be related to the degree of OS (72).

Selenium status in neonates is difficult to estimate based on the literature due to the variability of selenium sample matrix (e.g., serum and erythrocyte selenium concentration, activity of GPx, urinary selenium), reported outcomes, and outcome metrics (e.g., μg/L, μmol/L, ng/g Hb, μmol/mmol creatinine). The present study investigated neonatal SeS levels in addition to SeU levels, since SeS is reliable in evaluating short-term changes in the body, and SeU may be used for diagnosis and follow-up of Se deficiency. The oxidative stress caused by an imbalance between ROS and antioxidants may affect fetal development in the intrauterine period and may also induce long-term metabolic changes in these children (72, 73). An estimation of the impact of OS could be done by quantifying the levels of antioxidants such as Se (74).

The present study provided useful information about the status of serum/urinary selenium at birth in term neonates from mothers with hypertensive pathology. Se levels were evaluated within the 24 h after birth, because once babies are fed, these levels could be influenced by the Se content of breast milk and the additional nutritional support received by the mothers. Selenium supplementation in breastfeeding women is highly regarded, mostly in countries with Se dietary deficiencies such as Eastern Europe (30), and a daily intake of 85 µg Se was recommended during lactation by the European Food Safety Authority to meet an infant's recommended nutrient intake of 12 µg/day during the first six months of life (75). Our study conferred a clear image of the necessity and amplitude of Se supplementation even to term neonates and especially to those SGA to support their appropriate growth and development as well as to prevent a number of disorders and complications. Without any doubt, when feeding of these infants is based on infant formula, Se supplementation makes even greater sense. Since this is not routine practice in all countries, including Romania, and the optimal dose and length of selenium supplementation in neonates is not well-established, monitoring their Se levels is of outmost importance to overcome further associated disorders such as bronchopulmonary dysplasia, retinopathy, neuronal injury and altered thyroid function (28, 46).

We recognize some limitations of the study. First, the small sample size lowers the generalization of results and yet it provides real-world data that are important to understand contemporary maternal patterns and associated neonatal outcomes for focused intervention strategies aimed at supporting neonates for a proper development. Second, the study examined the absolute urine selenium levels without measuring the selenium-creatinine ratio. Since urinary creatinine levels can vary with hydration state, adjusting for creatinine can help to account for these variations and provide a more accurate measure of urine selenium status. Without this adjustment, the variations in creatinine levels could potentially mask true differences or similarities in urine selenium levels among study participants. Third, no biological specimens were collected from the mothers and the selenium concentrations during pregnancy were not available. Both maternal and fetal serum /urinary Se levels should be assayed in future studies, since complications due to oxidative stress in neonates are known to depend on maternal antioxidant conditions. Observation and analysis of the selenium status in the mother–fetus unit would offer additional information about the influence of maternal PIH on fetal development. This, in turn, could help to reduce the development of complications. Moreover, baseline, pre-pregnancy selenium concentrations were not available. Thus, we were not able to ascertain whether mothers delivering SGA infants had previously lower selenium concentrations compared with those delivering AGA infants.

## 5. Conclusions

Increased oxidative stress, like PIH and intrauterine growth restrictions, manifested postpartum as SGA, was associated with decreased levels of serum and urinary Se exhibited in newly born infants. Hence, selenium status of neonates proves to be a reliable indicator when evaluating the negative impact of PIH on their intrauterine development. Reduced selenium concentrations translate into a compromised antioxidant protection which could indicate the likelihood of further adverse outcomes along the infants' growth. Clinical markers of Se status could thus be of great value for tracking responses of individuals to selenium supplementation as part of health improvement and harm mitigation approaches. Future prospective, longitudinal studies will be required to elucidate the cause-and-effect relationship between Se status in both mother and child, PIH, and associated fetal outcomes.

## Data Availability

The raw data supporting the conclusions of this article will be made available by the authors, without undue reservation.
